# Supplementary material for: Modeling glioblastoma heterogeneity as a dynamic network of cell states
Source: Mol Syst Biol. 2021 Sep 16;17(9):e10105. doi: 10.15252/msb.202010105 (PMC8444284; doi:10.15252/msb.202010105)
Supplement: Supplementary file 6 — Source Data for Figure 5 [file MSB-17-e10105-s004.zip › Figure5A_sourcedata/GSEA_3017/nosigdb_stateE.GseaPreranked.1622795031093/FELDMAN_2020_G1.html]

Details for gene set FELDMAN\_2020\_G1[GSEA]

|  || Dataset | state63017 |
| Phenotype | NoPhenotypeAvailable |
| Upregulated in class | na\_neg |
| GeneSet | FELDMAN\_2020\_G1 |
| Enrichment Score (ES) | -0.7318381 |
| Normalized Enrichment Score (NES) | -4.0362716 |
| Nominal p-value | 0.0 |
| FDR q-value | 0.0 |
| FWER p-Value | 0.0 |
Table: GSEA Results Summary

  

Fig 1: Enrichment plot: FELDMAN\_2020\_G1      
 Profile of the Running ES Score & Positions of GeneSet Members on the Rank Ordered List

  

| PROBE | GENE SYMBOL | GENE\_TITLE | RANK IN GENE LIST | RANK METRIC SCORE | RUNNING ES | CORE ENRICHMENT || 1 | CTGF |  |  | 2 | 1.276 | 0.0113 | No |
| 2 | CYR61 |  |  | 3 | 1.169 | 0.0239 | No |
| 3 | IGFBP5 |  |  | 4 | 1.015 | 0.0348 | No |
| 4 | CALM2 |  |  | 191 | 0.303 | -0.1888 | No |
| 5 | SCG2 |  |  | 220 | 0.283 | -0.2199 | No |
| 6 | RAN |  |  | 298 | -0.257 | -0.3110 | No |
| 7 | PSIP1 |  |  | 319 | -0.263 | -0.3326 | No |
| 8 | HNRNPDL |  |  | 322 | -0.263 | -0.3322 | No |
| 9 | HNRNPA2B1 |  |  | 391 | -0.279 | -0.4121 | No |
| 10 | SIVA1 |  |  | 412 | -0.286 | -0.4334 | No |
| 11 | DNMT1 |  |  | 466 | -0.304 | -0.4948 | No |
| 12 | ILF2 |  |  | 497 | -0.313 | -0.5280 | No |
| 13 | GGH |  |  | 504 | -0.314 | -0.5319 | No |
| 14 | LSM3 |  |  | 530 | -0.323 | -0.5589 | No |
| 15 | HAT1 |  |  | 561 | -0.340 | -0.5918 | No |
| 16 | TROAP |  |  | 562 | -0.341 | -0.5882 | No |
| 17 | LSM5 |  |  | 568 | -0.342 | -0.5906 | No |
| 18 | HMGB3 |  |  | 577 | -0.348 | -0.5966 | No |
| 19 | DDX39A |  |  | 590 | -0.353 | -0.6074 | No |
| 20 | EXOSC8 |  |  | 630 | -0.376 | -0.6509 | No |
| 21 | RNASEH2A |  |  | 633 | -0.378 | -0.6493 | No |
| 22 | PHF19 |  |  | 648 | -0.391 | -0.6622 | No |
| 23 | TMEM106C |  |  | 654 | -0.397 | -0.6640 | No |
| 24 | GINS2 |  |  | 658 | -0.399 | -0.6634 | No |
| 25 | NUDT1 |  |  | 674 | -0.413 | -0.6772 | No |
| 26 | H2AFV |  |  | 684 | -0.423 | -0.6836 | No |
| 27 | RANBP1 |  |  | 697 | -0.427 | -0.6937 | No |
| 28 | ANP32B |  |  | 699 | -0.431 | -0.6903 | No |
| 29 | MZT1 |  |  | 711 | -0.437 | -0.6990 | No |
| 30 | RAD21 |  |  | 728 | -0.458 | -0.7135 | No |
| 31 | NUCKS1 |  |  | 744 | -0.471 | -0.7268 | Yes |
| 32 | PTMA |  |  | 745 | -0.475 | -0.7217 | Yes |
| 33 | TK1 |  |  | 747 | -0.480 | -0.7177 | Yes |
| 34 | MIS18A |  |  | 750 | -0.483 | -0.7149 | Yes |
| 35 | KNSTRN |  |  | 751 | -0.485 | -0.7097 | Yes |
| 36 | CDCA3 |  |  | 759 | -0.497 | -0.7129 | Yes |
| 37 | GMNN |  |  | 766 | -0.510 | -0.7147 | Yes |
| 38 | CMC2 |  |  | 771 | -0.518 | -0.7140 | Yes |
| 39 | DTYMK |  |  | 774 | -0.522 | -0.7109 | Yes |
| 40 | STMN1 |  |  | 778 | -0.529 | -0.7088 | Yes |
| 41 | CENPA |  |  | 782 | -0.538 | -0.7067 | Yes |
| 42 | RPA3 |  |  | 788 | -0.550 | -0.7069 | Yes |
| 43 | CDC20 |  |  | 793 | -0.562 | -0.7057 | Yes |
| 44 | HMGN2 |  |  | 795 | -0.564 | -0.7008 | Yes |
| 45 | HMGB1 |  |  | 797 | -0.567 | -0.6960 | Yes |
| 46 | TUBB4B |  |  | 804 | -0.573 | -0.6971 | Yes |
| 47 | CENPN |  |  | 805 | -0.574 | -0.6909 | Yes |
| 48 | USP1 |  |  | 806 | -0.575 | -0.6847 | Yes |
| 49 | PLK1 |  |  | 809 | -0.586 | -0.6809 | Yes |
| 50 | DEK |  |  | 812 | -0.591 | -0.6769 | Yes |
| 51 | DEPDC1 |  |  | 815 | -0.596 | -0.6730 | Yes |
| 52 | CDCA2 |  |  | 816 | -0.597 | -0.6665 | Yes |
| 53 | MCM7 |  |  | 817 | -0.599 | -0.6601 | Yes |
| 54 | LMNB1 |  |  | 818 | -0.606 | -0.6536 | Yes |
| 55 | AURKA |  |  | 824 | -0.623 | -0.6530 | Yes |
| 56 | CKAP2L |  |  | 826 | -0.625 | -0.6474 | Yes |
| 57 | CCDC34 |  |  | 827 | -0.626 | -0.6407 | Yes |
| 58 | DNAJC9 |  |  | 828 | -0.633 | -0.6339 | Yes |
| 59 | AURKB |  |  | 829 | -0.634 | -0.6271 | Yes |
| 60 | PCNA |  |  | 832 | -0.640 | -0.6226 | Yes |
| 61 | BUB3 |  |  | 834 | -0.641 | -0.6169 | Yes |
| 62 | CENPW |  |  | 835 | -0.643 | -0.6100 | Yes |
| 63 | CDKN3 |  |  | 836 | -0.653 | -0.6030 | Yes |
| 64 | ANP32E |  |  | 841 | -0.676 | -0.6006 | Yes |
| 65 | KIF22 |  |  | 842 | -0.678 | -0.5933 | Yes |
| 66 | BIRC5 |  |  | 843 | -0.679 | -0.5860 | Yes |
| 67 | CENPM |  |  | 844 | -0.682 | -0.5786 | Yes |
| 68 | ZWINT |  |  | 847 | -0.692 | -0.5736 | Yes |
| 69 | CENPH |  |  | 849 | -0.694 | -0.5674 | Yes |
| 70 | DHFR |  |  | 851 | -0.701 | -0.5610 | Yes |
| 71 | VRK1 |  |  | 852 | -0.711 | -0.5534 | Yes |
| 72 | NUF2 |  |  | 856 | -0.721 | -0.5493 | Yes |
| 73 | CENPK |  |  | 857 | -0.722 | -0.5415 | Yes |
| 74 | ANLN |  |  | 860 | -0.733 | -0.5361 | Yes |
| 75 | KIF4A |  |  | 861 | -0.736 | -0.5281 | Yes |
| 76 | FEN1 |  |  | 862 | -0.736 | -0.5202 | Yes |
| 77 | FBXO5 |  |  | 864 | -0.742 | -0.5134 | Yes |
| 78 | CCNB2 |  |  | 865 | -0.745 | -0.5054 | Yes |
| 79 | RRM1 |  |  | 866 | -0.749 | -0.4974 | Yes |
| 80 | NDC80 |  |  | 869 | -0.762 | -0.4916 | Yes |
| 81 | ORC6 |  |  | 870 | -0.766 | -0.4833 | Yes |
| 82 | CCNA2 |  |  | 872 | -0.770 | -0.4763 | Yes |
| 83 | KIF23 |  |  | 873 | -0.772 | -0.4680 | Yes |
| 84 | TMPO |  |  | 875 | -0.778 | -0.4608 | Yes |
| 85 | KIF20B |  |  | 876 | -0.779 | -0.4524 | Yes |
| 86 | CEP55 |  |  | 877 | -0.780 | -0.4440 | Yes |
| 87 | CKAP2 |  |  | 878 | -0.781 | -0.4356 | Yes |
| 88 | PTTG1 |  |  | 880 | -0.791 | -0.4283 | Yes |
| 89 | H2AFX |  |  | 882 | -0.799 | -0.4209 | Yes |
| 90 | UBE2S |  |  | 887 | -0.815 | -0.4170 | Yes |
| 91 | H2AFZ |  |  | 893 | -0.849 | -0.4140 | Yes |
| 92 | KPNA2 |  |  | 895 | -0.858 | -0.4060 | Yes |
| 93 | DBF4 |  |  | 896 | -0.864 | -0.3967 | Yes |
| 94 | SMC2 |  |  | 897 | -0.867 | -0.3873 | Yes |
| 95 | CENPE |  |  | 899 | -0.873 | -0.3792 | Yes |
| 96 | PBK |  |  | 900 | -0.880 | -0.3697 | Yes |
| 97 | SMC4 |  |  | 902 | -0.886 | -0.3614 | Yes |
| 98 | DUT |  |  | 903 | -0.887 | -0.3518 | Yes |
| 99 | CCNB1 |  |  | 906 | -0.895 | -0.3446 | Yes |
| 100 | CKS1B |  |  | 909 | -0.912 | -0.3372 | Yes |
| 101 | CKS2 |  |  | 911 | -0.922 | -0.3285 | Yes |
| 102 | GTSE1 |  |  | 912 | -0.941 | -0.3184 | Yes |
| 103 | CLSPN |  |  | 916 | -0.964 | -0.3117 | Yes |
| 104 | TYMS |  |  | 917 | -0.965 | -0.3013 | Yes |
| 105 | CENPU |  |  | 918 | -0.971 | -0.2908 | Yes |
| 106 | PRC1 |  |  | 919 | -0.980 | -0.2803 | Yes |
| 107 | MAD2L1 |  |  | 921 | -0.990 | -0.2709 | Yes |
| 108 | UBE2T |  |  | 922 | -0.994 | -0.2602 | Yes |
| 109 | DLGAP5 |  |  | 923 | -0.998 | -0.2494 | Yes |
| 110 | SPC25 |  |  | 924 | -1.033 | -0.2383 | Yes |
| 111 | HIST1H1E |  |  | 925 | -1.049 | -0.2270 | Yes |
| 112 | ESCO2 |  |  | 926 | -1.067 | -0.2155 | Yes |
| 113 | UBE2C |  |  | 928 | -1.108 | -0.2048 | Yes |
| 114 | NUSAP1 |  |  | 929 | -1.112 | -0.1928 | Yes |
| 115 | TPX2 |  |  | 930 | -1.124 | -0.1807 | Yes |
| 116 | NCAPG |  |  | 931 | -1.142 | -0.1684 | Yes |
| 117 | ASPM |  |  | 932 | -1.146 | -0.1561 | Yes |
| 118 | ATAD2 |  |  | 933 | -1.162 | -0.1436 | Yes |
| 119 | CENPF |  |  | 935 | -1.200 | -0.1319 | Yes |
| 120 | HIST1H1C |  |  | 936 | -1.220 | -0.1187 | Yes |
| 121 | HMGB2 |  |  | 937 | -1.238 | -0.1054 | Yes |
| 122 | CDK1 |  |  | 938 | -1.248 | -0.0920 | Yes |
| 123 | RRM2 |  |  | 940 | -1.403 | -0.0781 | Yes |
| 124 | MKI67 |  |  | 941 | -1.407 | -0.0629 | Yes |
| 125 | TOP2A |  |  | 942 | -1.417 | -0.0477 | Yes |
| 126 | HIST1H1B |  |  | 945 | -1.903 | -0.0296 | Yes |
| 127 | HIST1H4C |  |  | 946 | -2.752 | 0.0000 | Yes |
Table: GSEA details [plain text format]

  

Fig 2: FELDMAN\_2020\_G1: Random ES distribution      
 Gene set null distribution of ES for **FELDMAN\_2020\_G1**
